# Dentition of Early American Races

**Published:** 1886-05

**Authors:** 


### SELECTIONS.

### Dentition of Early American Races.

The 10th report of the Peabody Museum of American Archæology and Ethnology at Cambridge, Mass., contains, "Some notes upon Human Remains from the Caves of Coahuila, Mexico," by Cornelia A. Studley, of which the following abstract and quotations find a fitting place in the columns of a dental journal:

The collection was formed by Dr. Palmer, in 1880, the caves examined being situated in the limestone formation of the State of Coahuila, Mexico.

The first cave was discovered in 1865, by an Indian. It was then 150 feet long, 20 feet wide, and 12 feet high. Unfortunately the roof has fallen in and buried most of the remains left by the first treasure hunters. Dr. Palmer secured, however, four perfect crania and as many almost complete skeletons. From the second cave, about 75 feet square, reported to have contained in 1838, a great many mummies, only fifteen skulls and a few fragments were obtained. From the third, six crania, and from the fourth only one rewarded the explorer. The whole neighborhood was worked some twenty years since for saltpetre, and most of the mummies seem to have fed the fires of the saltpetre miners.

The bones from the various caves are well preserved. They indicate a muscular and strongly built people. None of the bones of the skeletons show marks of disease.

The average height may be estimated at 5 feet 3 inches. The palate is either hypsiloid or slightly elliptical in form. None are prognathic. Five males and two children are orthognathic, four males and one female mesognathic. There is no dental prognathism, for the incisors in those jaws in which they remain are set vertically in the sockets. A female skull has a small conical hyperostosis on the superior maxillary, midway between the lower margin of the orbit and the first molar. The lower jaw is noticeably slender. Its actual weight is less than a Californian, Peruvian or Moundbuilder's jaw of the same period of life. The height at the symphisis is always greater than at the last molar. The chin is prominent. The ramus is narrow and low, its coronoid slighly exceeding the condyloid process in height. The sigmoid notch is shallow. The condyle is small. The angle is everted in eleven of the fourteen jaws.

There is no anomaly of the teeth except a small supernumerary tooth between the central incisors of each of the two upper jaws. All the teeth are large and closely set. The incisors are vertical. The first upper molar shows traces of four cusps, but in all adult upper jaws, this molar is worn so as to expose the ivory, and in four instances a clean section of the tooth has been made. In one case no enamel is to be seen, the tooth is worn to the neck, and the pulp cavity exposed at this point, a condition Broca designates as exceptional. There has been an abscess at the root of the tooth with considerable alveolar absorption probably to be traced to the inflammation of attrition after the pulp was exposed. The condition of the teeth in all these crania points to the use of hard, coarse food. One hundred and eighty-nine teeth are retained in the sockets of the twenty-three upper jaws. The rest with few exceptions have fallen out post-mortem. Two per cent of the retained teeth are carious, and two per cent of the empty sockets show traces of inflammatory action about the roots of missing teeth.

The lower jaw has also large teeth set close together. Its first molar has commonly five cusps. The wear is downwards and outwards for all but the incisors, which wear horizontally. Some of the teeth of both upper and lower jaws are excavated by use. The fourteen lower jaws have retained 141 teeth, of which but one is carious. None of the sockets of the lower jaw point to an unhealthy condition of the missing teeth. In one upper jaw the teeth are worn flat nearly to the neck. Upon the right side the molars have been lost by disease, the alveoli are absorbed. The loss of these teeth has probably effected the wear of the second bicuspid, adjoining which has the posterior half of the crown is worn off obliquely upwards and backwards. Upon the left side is the first molar, whose extreme degree of wear has been already described. The foregoing observations indicate that the people who buried their dead in the caves of Coahuila were a strong built, muscular, long-headed race of medium height. Wisliczenus mentions a conjecture that they belonged to the tribe of the ancient Lipans.

The tables appended to these notes are admirable specimens of the exactness of modern scientific method.

Removing Microbes from Water.—From recent experiments of Prof. Frankland it appears that coke, spongy iron, animal charcoal, when reduced to particles small enough to pass through a sieve of forty meshes to the inch, and placed in columns six inches in height, were capable of completely removing micro-organisms from water filtered through them. This power was lost after being in use for a month, though all except animal charcoal continued to remove a considerable portion. By agitating a gramme of each of these substances with 50 c. c. of water for fifteen minutes it was found that the number of organisms could be greatly reduced, and in the case of coke could be removed altogether. R. B. D.

